# Association of spinal cord structure with cognition in hereditary spastic paraplegia type 5

**DOI:** 10.3389/fneur.2025.1639011

**Published:** 2026-01-02

**Authors:** Xuanyu Chen, Kunxin Lin, Liangliang Qiu, Ruying Yuan, Yusen Qiu, Jin Bi, Zhixian Ye, Wanjin Chen, Ning Wang, Ying Fu

**Affiliations:** 1 Department of Neurology, The First Affiliated Hospital, Fujian Medical University, Fuzhou, Fujian, China; 2 Department of Neurology, National Regional Medical Center, Binhai Campus of the First Affiliated Hospital, Fujian Medical University, Fuzhou, Fujian, China; 3 Fujian Key Laboratory of Molecular Neurology, Fujian Medical University, Fuzhou, Fujian, China

**Keywords:** brain, cognitive impairment, magnetic resonance imaging, spastic paraparesis, spinal cord

## Abstract

**Background and objectives:**

Cognitive deficits often accompany brain structural changes, but the association between spinal cord atrophy and cognitive function remains unclear. Spastic paraplegia type 5 (SPG5) is a subtype of hereditary spastic paraplegias (HSPs), caused by mutations in cholesterol metabolism genes, with radiological features of spinal cord atrophy. Our study aims to explore cognitive impairment in SPG5 and its relation to spinal cord structure.

**Methods:**

We conducted a prospective cross-sectional study with 30 HSPs patients and 47 healthy controls (HC), performing brain and spinal cord MRI and neuropsychological assessments. We investigated correlations between cognitive performance and MRI measurements.

**Results:**

Compared to HC, SPG5 patients exhibited significant impairments in the Benton Judgment of Line Orientation Test (*p* = 0.033), Controlled Oral Word Association Test (*p* = 0.028), and Symbol Digit Modalities Test (*p* = 0.023). SPG5 patients also demonstrated significant reductions in spinal cord and thoracic cord cross-sectional area (CSA), anterior-posterior (AP) diameter, and right-left (RL) total (All *p* < 0.01). Specifically, the thoracic cord RL total was positively correlated with scores on the Brief Visuospatial Memory Test - Revisited delayed recall (BVMT-R DR) and Montreal Cognitive Assessment (MoCA). The spinal cord RL total explained 81% of the variance in BVMT-R DR (*p* = 0.015) and 74% of the variance in MoCA (*p* = 0.027).

**Discussion:**

SPG5 patients demonstrate deficits in visuospatial perception, executive function, and information processing speed. Thoracic spinal cord RL total was positively associated with visual memory and global cognition.

## 1 Introduction

Hereditary Spastic Paraplegias (HSPs) are a category of rare hereditary neurodegenerative motor neuron diseases, with a prevalence of two to five cases per 100,000 individuals worldwide (1, 2). They are characterized by progressive age-dependent loss of corticospinal motor tract function (3), and exhibit genetic heterogeneity. Over 90 spastic paraplegia genes (SPGs) have been identified, including SPG1-SPG90B (1, 4, 5). Inheritance patterns vary, with autosomal dominant being the most common (70%) (6). Notably, clinical phenotypic variations can occur within the same SPG type or between different SPG types.

Spinal cord involvement is a common pathological substrate across several SPG genotypes. Neuroimaging studies frequently show cervical-predominant cord atrophy with tract-specific changes. In SPG4, cervical spinal cord cross-sectional area (CSA) is reduced with anteroposterior flattening. Lateral and dorsal column involvement is prominent, and these metrics are associated with disease severity on the Spastic Paraplegia Rating Scale (SPRS) (7, 8). In SPG5, total spinal cord area is reduced, most notably at thoracic levels (9, 10). In SPG11, longer disease duration correlates with more severe cord atrophy (11).

While HSPs are primarily characterized by motor symptoms like progressive spasticity and weakness in the lower limbs, non-motor symptoms are gaining recognition due to their potential response to treatment. Cognitive impairment in HSPs is particularly noteworthy due to its detrimental effects on overall quality of life, impacting employment, social relationships, and daily activities (12).

SPG5 stems from mutations in the *CYP7B1* gene, encoding oxysterol-7α-hydroxylase crucial for cholesterol metabolism (13). These mutations disrupt cholesterol metabolism, leading to the accumulation of 25- and 27-hydroxycholesterol in plasma and cerebrospinal fluid (CSF) (14). Despite associations between cholesterol metabolism abnormalities and cognitive impairment in diseases like Alzheimer’s disease (AD), multiple sclerosis (MS), and Huntington’s disease (HD) (15–17), the cognitive impairment pattern in SPG5 remains unclear. Previous research noted cognitive deficits in SPG4 patients across memory, attention, executive function, and language fluency domains (18, 19). In SPG11 patients, cognitive impairments primarily affect executive function, delayed recall, abstraction, and language (20–22). Yet, differences in cognitive impairment patterns between other HSP subtypes and SPG5 require further investigation.

Neurodegenerative diseases can affect sensorimotor control, closely tied to cognitive functions. Given the spinal cord’s role in transmitting sensorimotor signals from the brain to the peripheral nervous system, investigating whether spinal cord injuries could retrogradely impair cognitive functions is relevant. Our previous study found spinal cord atrophy in SPG5 patients (9). Exploring the relationship between spinal cord structure and cognitive impairment in SPG5 could provide insight into this issue.

In this cross-sectional cohort study, we employed the modified Chinese version of Minimal Assessment of Cognitive Function in Multiple Sclerosis (MACFIMS) to evaluate cognitive functions in HSPs patients (23). The reliability and validity of this cognitive assessment scale have been previously validated in our spinocerebellar ataxia type 3 (SCA3) patient cohort (24). Our primary aim is to assess cognitive impairment patterns in SPG5 and examine the link between cognitive deficits in specific domains and spinal cord structures. Additionally, we compare neuropsychological differences between SPG5 and other HSP subtypes, and investigate associations between cognitive impairments and brain structures among HSP patients.

## 2 Materials and methods

### 2.1 Participants and design

This project made use of data collected from a prospective cohort study (ClinicalTrials.gov Identifier: NCT04006418). The study was approved by the ethics committee of the first affiliated hospital of Fujian Medical University, Fujian, China (project approval number MRCTA, ECFAH of FMU [2019]194), and written informed consent was obtained from all participants.

The study design of this HSPs cohort study was detailed in a previous protocol (25). In summary, this cohort study collected comprehensive data from HSPs patients across six key aspects, encompassing clinical signs, genetic spectrum, cognitive competence, MRI features, as well as potential biochemical indicators and nerve electrophysiological factors. The project included 30 HSPs patients, comprising 25 clinically and genetically confirmed cases (11 SPG4, 10 SPG5, 2 SPG11, 1 SPG30 and 1 SPG76) and five clinically suspicious cases (e.g., exhibiting progressive spastic gait disorder, bilateral lower limb spasm and hyperreflexia, among others). Genomic DNA was analyzed through multiple ligation-dependent probe amplification (MLPA) in conjunction with whole exome sequencing (WES). All enrolled patients fulfilled the predefined exclusion criteria, which encompassed subacute combined degeneration, vitamin E deficiency, syphilis, AIDS, tropical spastic paraplegia, spinocerebellar ataxia, Friedreich’s ataxia and any structural abnormalities or severe lesions that could potentially cause spastic paraplegia. The 47 HC included in this study with no previous history of neurological dysfunction and with normal findings on neurological examination were recruited in the same hospital. All participants with psychiatric disorders, other diseases or disorders that may affect cognitive impairment, and those who did not cooperate with the cognitive assessment were excluded.

### 2.2 Cognitive assessment

All participants (30 HSPs and 47 HC) underwent assessment by the same neurologist in a standardized manner. The neurologist conducting the evaluations remained blinded to the clinical conditions of the participants. The assessments were performed during daytime, in a quiet room, and in a fixed order, following the recommendations of the consensus panel.

Total cognitive function was assessed using the MMSE and MoCA. Moreover, we utilized the modified Chinese version of MACFIMS scale to evaluate performance in five cognitive domains (25). Specifically, Verbal memory was gauged through the immediate and delayed recall scores of the California Verbal Learning Test-second Edition (CVLT-II). Visual memory was evaluated using the immediate and delayed recall scores of the Brief Visuospatial Memory Test-revised (BVMT-R). Visuospatial perception was examined with the Benton Judgment of Line Orientation Test (JLO). Executive functions were measured using the Controlled Oral Word Association Test (COWAT). Information processing speed was assessed using the Symbol Digit Modalities Test (SDMT) and the Paced Auditory Serial Addiction Test (PASAT) 3 and 2-sand versions.

Furthermore, the Hamilton Depression Rating Scale (HAMD) was employed to assess depression, the Hamilton Anxiety Rating Scale (HAMA) for anxiety evaluation, and the Fatigue Scale-14 for fatigue assessment. Sleep quality was evaluated using the Pittsburgh Sleep Quality Index (PSQI), and daytime sleepiness was assessed using the Epworth Sleepiness Scale (ESS). The entire battery of tests required 90–100 min of face-to-face testing time.

Patients with test scores deviating over 1.5 SDs from the HC mean were deemed to have abnormal function in the related cognitive domain. Those with abnormalities in two or more domains were categorized as having cognitive impairment (CI) (23), while others were classified as cognitively preserved (CP). Furthermore, to standardize the scores of different cognitive assessment scales across the study and to compare the severity of cognitive impairment in patients with HSPs, we calculated the ratio of various cognitive scores for each HSPs-CI patient to the average HC score decrease of more than 1.5 SDs. A ratio greater than or equal to 1 corresponds to an abnormality in this cognitive domain that is not evident in HSPs patients, and a ratio less than 1 corresponds to the abnormal functioning of this cognitive domain being significant.

### 2.3 MRI data acquisition and analysis

Certain subjects underwent MRI examinations using a 3 T MRI Scanner (Siemens; Munich, Germany) equipped with a 20-channel head–neck coil and a 24-channel spine-array coil. The acquired sequences included: (1) Axial T2-weighted imaging (T2WI), T2-fluid-attenuated inversion recovery (FLAIR), and sagittal 3D T1-weighted imaging (T1WI) of the entire brain; (2) Sagittal 3D-T2WI and 3D-T1WI of the cervical and thoracic spinal cord (25).

To quantify the volumes of distinct regions, encompassing grey matter (GM, sum of voxels classified as GM), white matter (WM, sum of voxels classified as WM), and CSF, 3D-T1WI images underwent meticulous examination and processing (9). This analysis was conducted utilizing the Computational Anatomy Toolbox V.12 (CAT V.12, http://www.neuro.uni-jena.de/cat/), an add-on for Statistical Parametric Mapping V.12 (SPM V.12, http://www.fil.ion.ucl.ac.uk/spm), implemented within the MATLAB (R2013b, the MathWorks; Natick) environment. Additionally, the total intracranial volume (TIV) was defined as the sum of all voxels classified as GM, WM, or CSF.

Each cervical-thoracic spinal segment was acquired through the utilization of Compose software on the SIEMENS workstation. The Spinal Cord Toolbox (V.4.01) will be employed for segmenting and extracting the morphological metrics of the spinal cord at various vertebral levels, spanning from C1 to T9. These metrics will encompass CSA and sectional diameter (anterior–posterior AP, right–left RL, and the AP/RL ratio) (9).

### 2.4 Statistical analysis

Continuous variables are expressed using the median (IQR). Categorical data were expressed as percentages (%). The Kolmogorov–Smirnov test was employed to assess variable normality. Non-parametric continuous variables were compared using the Mann–Whitney U test (for 2 groups), and independent samples t-test were utilized for normally distributed continuous variables. Comparisons between categorical variables were conducted using the χ^2^ test (Fisher’s exact test when expected values are <5). Pearson or Spearman rank correlation coefficients were used to assess the correlation between variables.

Furthermore, linear regression models were employed with BVMT-R DR or MoCA as the dependent variable and spinal cord morphometric indices as independent variables to assess the extent to which spinal measures explained variance in cognition. We then re-estimated these models, separately including BPF and disease duration as covariates, to assess the associations between spinal morphometric indices and BVMT-R DR or MoCA.

Data analysis was performed using SPSS Statistics (version 26; IBM, USA). Results were considered statistically significant at the level of *p* < 0.05.

## 3 Results

### 3.1 Demographic data

A total of 30 HSPs patients and 47 HC were recruited in the cohort. Five clinically suspected cases were excluded owing to lack of genetic diagnosis. One patient was excluded due to an absence of neuropsychological data. Ultimately, 24 genetically confirmed HSPs patients were included in this analysis.

The age of examination of the 24 HSPs patients (10 SPG4, 10 SPG5, 2 SPG11, 1 SPG30, and 1 SPG76) ranged from 28 to 50 years (median 37 years); 8 (33.3%) were women. All patients with HSPs exhibited clinical symptoms, with median age at disease onset was 22 years (IQR 11–40 years) suffered a median disease duration of 12 years (IQR 5–15 years). The age at examination of the 47 HC was similar to that of patients with HSPs, with an age range of 26 to 45 (median 33); 24 (51.1%) were women. There were no significant differences in the age or gender of HSPs and HC.

For each HSPs group, no significant differences were found between SPG5 patients and other-HSPs patients (10 SPG4, 2 SPG11, 1 SPG30 and 1 SPG76) by age. However, the proportion of women (*p* = 0.002) was higher, the age of onset (*p* = 0.048) was earlier and the disease duration (*p* = 0.014) was longer in SPG5 patients compared with other-HSPs patients. The details of demographic and clinical characteristics of SPG5 and other-HSPs are presented in Table 1.

**Table 1:** Demographic and clinical characteristics of HSPs and HC.

Characteristic	HSPs, median (IQR) or No. (%)			HC (*N* = 47)	*P*-value^a^
	Total (*N* = 24)	SPG5 (*N* = 10)	other-HSPs (*N* = 14)		
Age, years	37 (28–50)	33 (30–44)	43 (22–51)	33 (26–45)	0.688
Female	8 (33.3)	7 (70.0)	1 (7.1)^**^	24 (51.1)	**0.002**
Education, years	12 (9–15)^*^	12 (9–16)	10.5 (9–12)^*^	16 (9–16)	0.192
0<~≤9 years	10 (41.7)	3 (30.0)	7 (50.0)	13 (27.6)	
9<~≤12 years	8 (33.3)	3 (30.0)	5 (35.7)	6 (12.8)	
>12 years	6 (25.0)	4 (40.0)	2 (14.3)	28 (59.6)	
Right handedness	30 (100.0)	10 (100.0)	14 (100.0)	47 (100.0)	1.000
Disease duration, years	12 (5–15)	15 (12–22)	6 (4–13)	-	**0.014**
Age at onset, years	22 (11–40)	13 (10–28)	34 (16–45)	-	**0.048**
MMSE	30.0 (28.0–30.0)^b^	30.0 (29.0–30.0)	29.0 (27.5–30.0)^c^	30.0 (29.0–30.0)^d^	0.115
MoCA	27.0 (26.0–28.0)^*,^^b^	28.0 (25.8–30.0)	27.0 (25.5–28.0)^**,^^c^	29.0 (28.0–30.0)^e^	0.166

HSPs, Hereditary Spastic Paraparesis; SPG5, spastic paraplegia type 5; HC, Healthy Control; MMSE, Mini-Mental State Examination; MoCA, Montreal Cognitive Assessment; IQR, interquartile range. Other-HSPs refer to genetically confirmed other HSPs subtypes except SPG5, include SPG4, SPG11, SPG30 and SPG76. Analyses were undertaken to examine differences in age, education, MMSE, and MoCA among the HSPs total, SPG5, and other-HSP groups relative to the HC, signified by * for *p* < 0.05 and ** for *p* ≤ 0.01. Statistical difference was determined by Mann–Whitney U and independent samples *t*-test.

a SPG5 vs other-HSPs.

b *n* = 23.

c *n* = 13.

d *n* = 18.

e *n* = 27.

### 3.2 Cognitive impairment in HSPs

For general cognitive assessment, HSPs patients had lower education levels (*p* = 0.031) and MoCA scores (*p* = 0.012) than HC (Table 1). We next investigated the five cognitive domains, including verbal memory (CVLT-II), visual memory (BVMT-R), visuospatial perception (JLO), executive function (COWAT) and information processing speed (SDMT, PASAT 2 s and PASAT 3 s).

We observed that CVLT-II, JLO, COWAT, SDMT and PASAT 3 s scores decreased significantly in patients with HSPs (All *p* < 0.05), while there was no significant difference in BVMT-R between HSPs patients and HC. Specifically, for the CVLT-II scores of HSPs patients, the scores of short-term storages (I to V-test and total of five learning trials), short-term and long-term delayed free recall (SDFR and LDFR), short-term and long-term delayed cued recall (SDCR and LDCR), and total of delayed recall (CVLT-II DR) were significantly reduced (Table 2).

**Table 2:** Cognitive test scores of HSPs and HC.

Cognitive tests	HSPs, median (IQR)			HC (*N* = 47)	*P*-value^a^
	Total (*N* = 24)	SPG5 (*N* = 10)	other-HSPs (*N* = 14)		
Verbal memory					
CVLT-II					
I	4.0 (3.0–5.8)^**^	5.5 (3.8–6.3)	3.5 (3.0–5.0)^***^	6.0 (4.0–8.0)	0.089
II	7.0 (6.0–8.0)^**^	8.0 (6.8–8.5)	6.5 (4.0–7.0)^**^	9.0 (7.0–10.0)	**0.016**
III	7.5 (6.3–9.8)^***^	8.5 (7.0–11.5)	7.0 (6.0–8.3)^***^	11.0 (9.0–13.0)	0.084
IV	9.5 (7.3–11.8)^***^	10.0 (7.0–12.3)	9.0 (7.8–11.0)^***^	13.0 (10.0–14.0)	0.250
V	10.0 (8.3–12.8)^**^	10.5 (9.5–14.0)	9.5 (8.0–12.3)^**^	13.0 (11.0–14.0)	0.350
TL	38.0 (31.0–45.5)^***^	42.5 (35.8–51.0)	35.0 (29.8–43.3)^***^	51.0 (40.0–59.0)	0.070
SDFR	8.5 (7.0–11.0)^***^	9.5 (6.8–13.3)	7.5 (6.5–11.0)^**^	11.0 (10.0–14.0)	0.286
SDCR	10.0 (7.0–11.8)^**^	10.0 (9.5–12.5)	8.0 (5.0–11.3)^***^	12.0 (9.0–14.0)	0.099
LDFR	9.0 (6.0–12.8)^**^	11.0 (8.0–13.3)	7.5 (5.0–12.0)^**^	12.0 (10.0–14.0)^b^	0.104
LDCR	9.5 (6.3–12.0)^**^	10.5 (8.5–13.3)	7.5 (4.8–11.3)^***^	12.0 (10.0–14.0)	0.097
DR	36.5 (23.5–46.5)^***^	40.0 (33.8–52.3)	31.5 (20.8–44.8)^***^	47.0 (39.8–56.0)^b^	0.111
FR insert	3.0 (1.0–4.0)^***^	2.0 (0.8–4.3)	3.0 (1.0–4.5)^**^	1.0 (0.0–2.0)	0.625
CR insert	1.5 (1.0–6.0)^**^	1.0 (0.8–2.3)	2.5 (1.0–7.0)^**^	1.0 (0.0–2.0)	0.192
All insert	7.0 (3.5–12.8)^***^	7.0 (4.3–9.3)^*^	7.0 (2.8–14.8)^**^	2.0 (1.0–7.0)	0.576
All repeat	4.5 (2.3–6.8)	3.5 (1.8–6.3)	5.0 (2.3–8.3)	5.0 (2.0–8.0)	0.488
LDR correct	14.5 (13.0–16.0)	15.0 (13.0–16.0)	14.5 (12.3–15.3)	15.0 (13.0–16.0)	0.437
Visual memory					
BVMT-R					
I	5.0 (2.5–9.5)	8.0 (3.5–9.0)	4.0 (2.0–10.0)	6.0 (4.0–10.0)	0.551
II	10.0 (6.5–12.0)	10.0 (8.0–10.5)	9.0 (6.0–12.0)	10.0 (8.0–12.0)	0.725
III	11.0 (6.5–12.0)	11.0 (9.5–12.0)	11.0 (6.0–12.0)	12.0 (10.0–12.0)	0.585
TL	26.0 (16.5–32.0)	29.0 (21.0–31.5)	22.0 (13.5–32.5)	26.0 (22.0–34.0)	0.585
DR	10.0 (8.5–12.0)	10.0 (10.0–12.0)	10.0 (7.5–12.0)	12.0 (10.0–12.0)	0.666
Recognition	12.0 (12.0–12.0)	12.0 (12.0–12.0)	12.0 (12.0–12.0)	12.0 (12.0–12.0)	0.931
Visuospatial perception					
JLO	21.0 (13.0–25.0)^**^	20.0 (13.0–25.3)^*^	21.0 (14.8–25.0)^*^	24.0 (21.0–28.0)	0.977
Executive function					
COWAT	10.0 (9.0–14.0)^***^	13.5 (9.0–17.5)^*^	9.5 (8.3–14.0)^***^	17.0 (13.0–22.0)	0.212
Information processing speed					
SDMT	44.0 (38.0–58.0)^***,^^c^	45.0 (35.5–60.0)^*^	44.0 (38.5–56.5)^**,^^d^	62.0 (49.0–69.0)	0.763
PASAT 3 s	52.0 (35.0–55.0)^*,^^c^	52.0 (34.5–58.3)	52.0 (40.0–54.5)^*,^^d^	54.0 (49.0–58.0)	0.605
PASAT 2 s	37.0 (27.5–48.3)^e^	33.0 (27.5–49.0)	40.0 (27.3–48.8)^f^	41.0 (33.0–52.0)	0.832

HSPs, Hereditary Spastic Paraparesis; SPG5, spastic paraplegia type 5; HC, Healthy Control; CVLT-II, California Verbal Learning Test-second Edition; TL, total learning; SDFR, short-delay free recall; SDCR, short-delay cued recall; LDFR, long-delay free recall; LDCR, long-delay cued recall; DR, delay recall; FR insert, free recall insert number; CR insert, cued recall insert number; LDR correct, long-delay recognition (correct number); BVMT-R, Brief Visuospatial Memory Test -revisited; JLO, Benton Judgment of Line Orientation Test; COWAT, Controlled Oral Word Association Test; SDMT, Symbol Digit Modalities Test; PASAT 2s and 3s, paced auditory serial addition task 2-second and 3-second; IQR, interquartile range. Other-HSPs refer to genetically confirmed other HSPs subtypes except SPG5, include SPG4, SPG11, SPG30 and SPG76. Analyses were undertaken to examine differences in each cognitive indicators among the HSPs total, SPG5, and other-HSP groups relative to the HC, signified by * for *p* < 0.05, ** for *p* ≤ 0.01 and *** for *p* ≤ 0.001s. Statistical difference was determined by Mann-Whitney U and independent samples *t*-test.

a SPG5 vs other-HSPs.

b *n* = 46.

c *n* = 23.

d *n* = 13.

e *n* = 22.

f *n* = 12.

**Figure 1:**
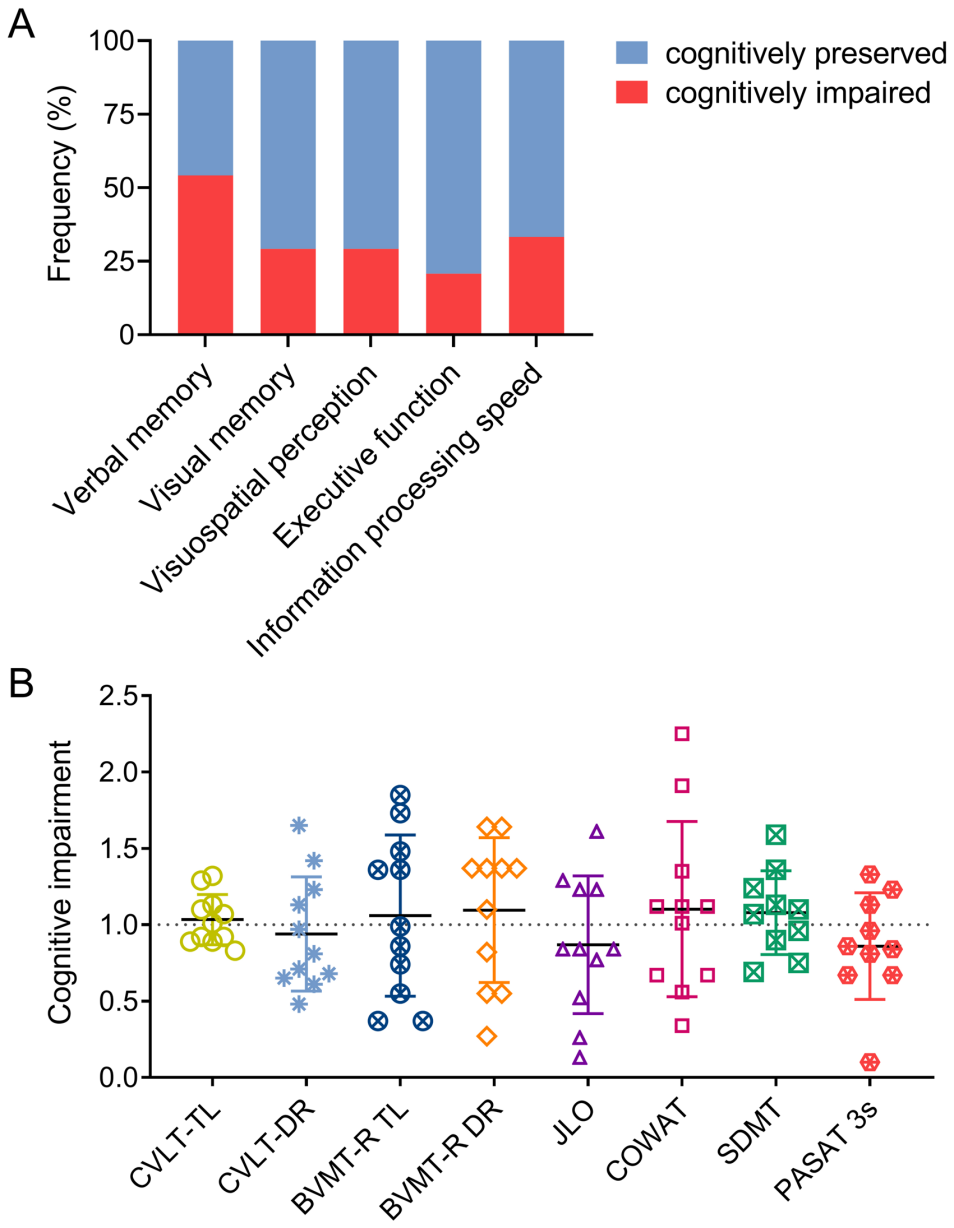
Cognitive impairment frequency and severity in HSPs patients. **(A)** The frequency of cognitive impairment (red histogram) and cognitive preservation (blue histogram) in the five cognitive domains of verbal memory, visual memory, visuospatial perception, executive function, and information processing speed among HSPs patients. **(B)** The depth of impairment in CVLT-II TL, CVLT-II DR, BVMT-R TL, BVMT-R DR, JLO, COWAT, SDMT, and PASAT 3 s scores among HSPs-CI patients. The bars denote the mean value ± SD. CVLT-II TL, California Verbal Learning Test-second Edition total learning; CVLT-II DR, California Verbal Learning Test-second Edition delay recall; BVMT-R TL, Brief Visuospatial Memory Test -revisited total learning; BVMT-R DR, Brief Visuospatial Memory Test -revisited delay recall; JLO, Benton Judgment of Line Orientation Test; COWAT, Controlled Oral Word Association Test; SDMT, Symbol Digit Modalities Test; PASAT 3 s, paced auditory serial addition task 3-s.

In general, about 45.8% of HSPs patients showed cognitive impairment. According to the five cognitive domains, 54.2% of patients had abnormal verbal memory function, 29.2% had abnormal visual memory function, 29.2% had abnormal spatial vision, 20.8% had abnormal executive function, and 33.3% had impaired information processing speed (Figure 1A). The CVLT-II DR, JLO and PASAT 3 s scores decreased significantly in patients with HSPs-CI, with JLO and PASAT 3 s showed the most pronounced decline (Figure 1B).

In addition, psychological scales assessed between patients with HSPs and HC, such as HAMA, HAMD, fatigue scale, PSQI, and ESS, did not showed significant differences (Supplementary Table 1).

### 3.3 Cognition comparison between SPG5 and other HSPs subtypes except SPG5 (other-HSPs)

For cognitive impairment mode of SPG5, there were no significant differences in the years of education, MMSE and MoCA between SPG5 patients and other-HSPs patients (Table 1). Assessment of five cognitive domains finds patients with SPG5 had lower JLO (*p* = 0.033), COWAT (*p* = 0.028) and SDMT (*p* = 0.023) scores compared with HC (Table 2). We did not find difference between SPG5 and HC in other cognitive assessments, including all tests in CVLT-II, BVMT-R, PASAT 2 s and 3 s. Besides, Patients with SPG5 had lower ESS (*p* = 0.041) scores, compared with HC (Supplementary Table 1).

Regarding the cognitive impairment pattern in individuals with other-HSPs, compared to HC, those with other-HSPs had shorter education duration (*p* = 0.013) and lower MoCA scores (*p* = 0.003) (Table 1). Assessment across five cognitive domains revealed that patients with other-HSPs exhibited lower scores in CVLT-II, JLO, COWAT, SDMT, and PASAT 3 s (All *p* < 0.05) compared to HC (Table 2). Additionally, psychiatric scale assessments indicated higher Fatigue scale scores (*p* = 0.038) in individuals with other-HSPs compared to HC (Supplementary Table 1).

This study further explored the differences in cognitive impairment patterns between patients with SPG5 and patients with other-HSPs. There was no significant difference in BVMT-R, JLO, COWAT, SDMT and PASAT 3 s or 2 s in SPG5 patients compared with other-HSPs patients. However, it is worth noting that the CVLT-II II-test (*p* = 0.016) score of SPG5 patients were higher compared to other-HSPs patients (Table 2). No significant differences were observed in psychiatric scale scores between SPG5 patients and other-HSPs patients (Supplementary Table 1).

### 3.4 Cerebral and spinal cord morphometry differences between HSPs and HC

**Figure 2:**
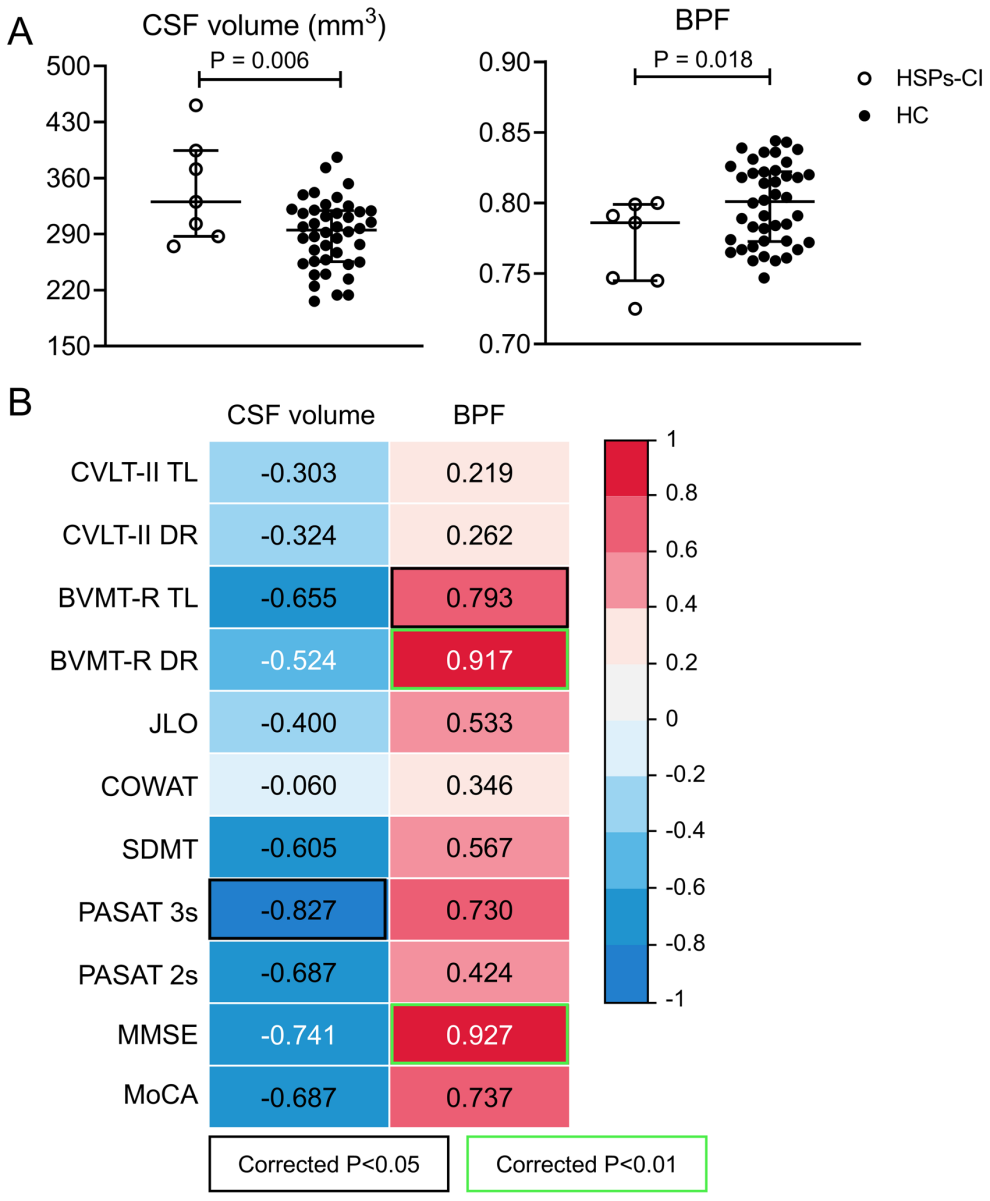
Changes in cerebral volume in HSPs-CI patients and its correlation with cognitive test scores. **(A)** CSF volume and BPF in HSPs-CI patients (*n* = 7, hollow circles) and HC (*n* = 42, solid circles). Statistical differences were assessed using independent samples t-test. The bars represent the median with IQR. **(B)** Correlation analysis of CSF volume and BPF with cognitive function in HSPs-CI patients, including test scores of CVLT-II, BVMT-R, JLO, COWAT, SDMT, PASAT 3 s, PASAT 2 s MMSE and MoCA. Results presented in terms of Pearson (blank text) or Spearman (white text) correlation coefficient (r). HSPs-CI, hereditary spastic paraparesis-cognitive impairment; HC, Healthy Control; CSF, Cerebrospinal fluid; BPF, brain parenchymal fraction; CVLT-II TL, California Verbal Learning Test-second Edition total learning; CVLT-II DR, California Verbal Learning Test-second Edition delay recall; BVMT-R TL, Brief Visuospatial Memory Test -revisited total learning; BVMT-R DR, Brief Visuospatial Memory Test -revisited delay recall; JLO, Benton Judgment of Line Orientation Test; COWAT, Controlled Oral Word Association Test; SDMT, Symbol Digit Modalities Test; PASAT 2 s and 3 s, paced auditory serial addition task 2-s and 3-s; MMSE, Mini-Mental State Examination; MoCA, Montreal Cognitive Assessment.

To evaluate brain structural changes in patients with HSPs-CI. Quantitative brain MRI was used to evaluate seven patients with HSPs-CI (2 SPG4, 3 SPG5 and 2 SPG11) and 42 HC (9). Compared with HC, CSF volume (*p* = 0.006) increased and brain parenchymal fraction (BPF, i.e., [GMV + WMV]/TIV, *p* = 0.018) decreased in HSPs -CI patients (Figure 2A). There was no significant difference in TIV, GMV, WMV, GMV/TIV and WMV/TIV between HSPs-CI and HC (Supplementary Figure 1).

**Figure 3:**
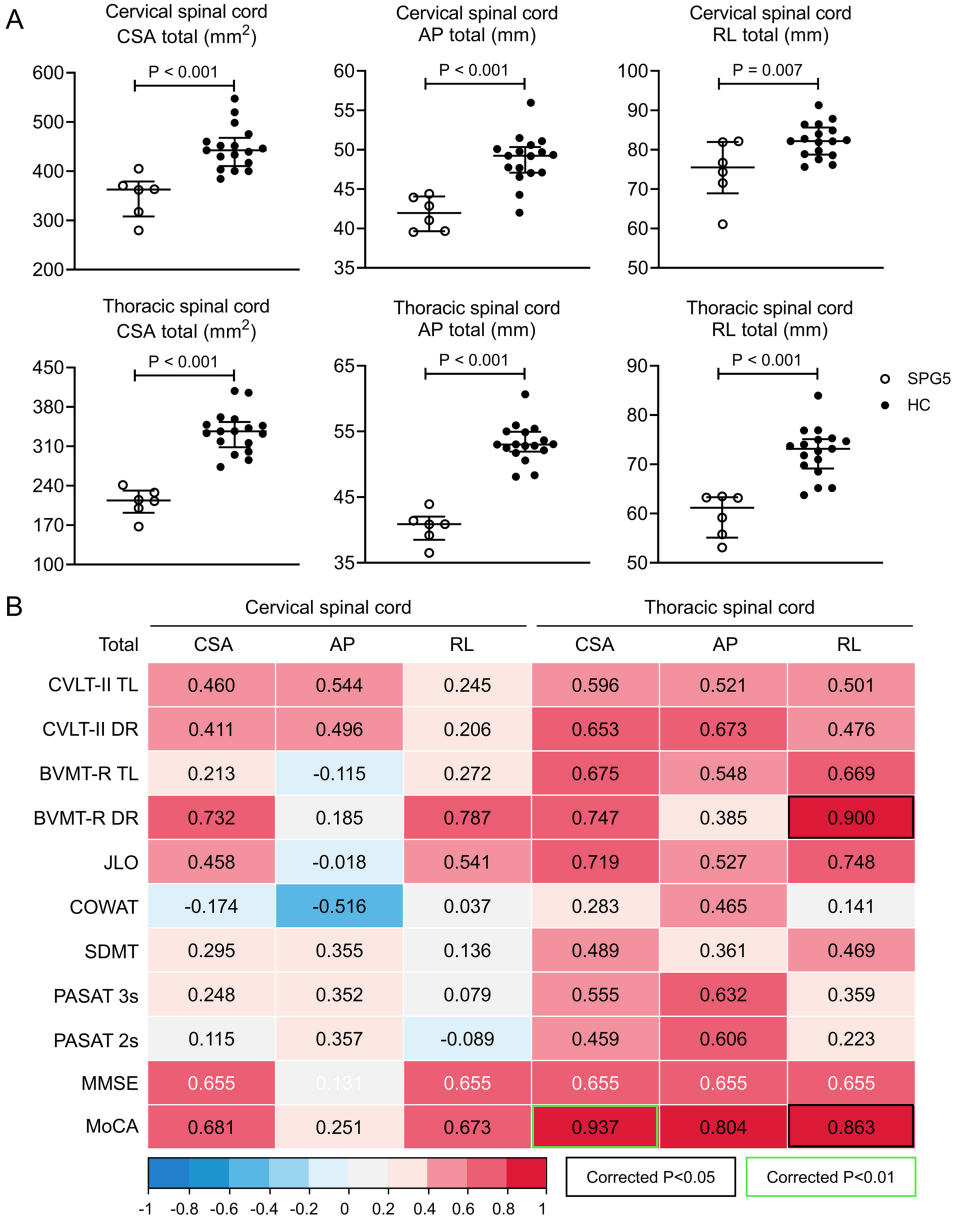
Changes in spinal cord cross-sectional morphometry in SPG5 patients and its correlation with cognitive test scores. **(A)** Comparison of cervical and thoracic spinal cord CSA total, AP total, RL total between SPG5 patients (*n* = 6, hollow circles) and HC (*n* = 17, solid circles). Statistical differences were examined through independent samples t-test. The bars represent the median with IQR. **(B)** Correlation analysis between cervical spinal cord CSA total, AP total, RL total, and cognitive function (including CVLT-II, BVMT-R, JLO, COWAT, SDMT, PASAT 3 s, PASAT 2 s MMSE and MoCA) in SPG5 patients. Results presented in terms of Pearson (blank text) or Spearman (white text) correlation coefficient (r). SPG5, spastic paraplegia type 5; HC, Healthy Control; CSA, cross-section area; AP, anterior to posterior diameter of spinal cord; RL, right to left diameter of spinal cord; CVLT-II TL, California Verbal Learning Test-second Edition total learning; CVLT-II DR, California Verbal Learning Test-second Edition delay recall; BVMT-R TL, Brief Visuospatial Memory Test -revisited total learning; BVMT-R DR, Brief Visuospatial Memory Test -revisited delay recall; JLO, Benton Judgment of Line Orientation Test; COWAT, Controlled Oral Word Association Test; SDMT, Symbol Digit Modalities Test; PASAT 2 s and 3 s, paced auditory serial addition task 2-s and 3-s; MMSE, Mini-Mental State Examination; MoCA, Montreal Cognitive Assessment.

Previous research by our team found significant cervical and thoracic spinal cord atrophy in patients with SPG5 compared to HC (9). In this study, we quantified cervical and thoracic spinal cord cross-sectional morphometry in six patients with SPG5. Similarly, the total of CSA, AP and RL in the cervical and thoracic spinal cord of SPG5 patients (All *p* < 0.01) were significantly smaller than that of HC (Figure 3A).

### 3.5 Correlation of cognitive impairment with cerebral and spinal cord cross-sectional morphometry

Subsequently, we evaluated the relationship between CSF volume, BPF, and cognitive scores in HSPs-CI patients (*N* = 7). CSF volume in HSPs-CI patients was negatively correlated with PASAT 3 s (Pearson’s *r* = −0.827, *p* = 0.022). BPF was positively correlated with BVMT-R TL (Pearson’s *r* = 0.793, *p* = 0.033), BVMT-R DR (Spearman’s *r* = 0.917, *p* = 0.004) and MMSE (Spearman’s *r* = 0.927, *p* = 0.003) (Figure 2B).

In addition, we further investigated the correlation between cervical and thoracic spinal cord cross-sectional morphometrics and cognition in patients with SPG5 (*N* = 6). For the cervical spinal cord, CSA total, AP total, and RL total showed no significant correlations with scores on the modified Chinese version of the MACFIMS, MMSE and MoCA (Figure 3B). For the thoracic cord, AP total was positively correlated with MoCA (Pearson’s *r* = 0.937, *p* = 0.006); RL total was positively correlated with BVMT-R DR (Pearson’s *r* = 0.900, *p* = 0.014) and MoCA (Pearson’s *r* = 0.863, *p* = 0.027) (Figure 3B). Linear regression showed that thoracic spinal cord RL total explained 81% of the total variance in BVMT-R DR scores (*p* = 0.015), and 74% of the total variance in MoCA scores (*p* = 0.027). There was no significant difference between the total AP of thoracic spinal cord and MoCA in linear regression.

In SPG5 (*N* = 6), global brain volumetrics (TIV, GMV, WMV, CSF, BPF) were not significantly associated with BVMT-R DR or MoCA (Supplementary Table 2). With BPF included as a covariate, thoracic RL total remained positively associated with BVMT-R DR (B = 0.218, 95% CI 0.068 to 0.367, *p* = 0.019) and MoCA (B = 0.329, 95% CI 0.105 to 0.554, *p* = 0.019), and thoracic CSA total remained associated with MoCA (B = 0.059, 95% CI 0.021 to 0.096, *p* = 0.015) (Supplementary Table 3). Cervical AP total correlated negatively with disease duration (Pearson’s *r* = −0.886, *p* = 0.019) (Supplementary Table 4). In models additionally adjusting for duration, thoracic RL total remained positively associated with BVMT-R DR (B = 0.220, 95% CI 0.109 to 0.332, *p* = 0.008), while its association with MoCA did not reach significance. By contrast, thoracic CSA total remained positively associated with MoCA after adjustment (B = 0.060, 95% CI 0.020 to 0.100, *p* = 0.017) (Supplementary Table 3).

## 4 Discussion

In this cohort study, we used the modified Chinese version of the MACFIMS scale to assess cognitive impairment in HSPs subtypes and investigate its association with brain and spinal cord imaging changes. We found that SPG5 patients exhibited cognitive impairment primarily in visuospatial perception, executive function, and information processing speed. Interestingly, the severity of impairment in these domains was not linked to spinal cord structure. However, decreased thoracic spinal cord RL total correlated with lower scores in visual memory (delayed recall) and MoCA. Compared to HC, other-HSPs patients (SPG4, SPG11, SPG30 and SPG76) showed impairment in language memory, visuospatial perception, executive function, and information processing speed, while SPG5 patients had better language memory function. MRI findings revealed larger CSF volume and smaller BPF in HSPs cognitive impairment (CI) patients compared to HC. Correlation analysis indicated that higher CSF volume correlated with lower information processing speed, while lower BPF correlated with decreased visual memory (short-term storage and delayed recall) and MMSE.

Cognitive impairment has been documented across several SPG genotypes. For SPG4, converging evidence indicates prominent executive dysfunction with accompanying deficits in attention, memory (including working and verbal memory), and verbal fluency (18, 19, 26). For SPG11, studies consistently report executive dysfunction with additional impairments in delayed recall, abstraction, verbal fluency, attention and memory (18, 20–22). By contrast, evidence for SPG5 remains limited. In one series of four patients, lower phonemic/category fluency and RAVLT A6/A7 scores relative to controls indicated possible executive and episodic memory involvement (18). Against this background, our study systematically profiled five domains in SPG5 (verbal memory, visual memory, visuospatial perception, executive function, and information processing speed). Relative to HC, SPG5 showed worse visuospatial perception, executive function, and information processing speed with preserved verbal memory. Compared with other-HSPs, SPG5 showed comparable performance in visual memory, visuospatial perception, executive function, and information processing speed, while verbal memory was relatively preserved.

The impact of spinal cord atrophy on cognition remains unclear. Consistent with prior studies (9, 10), SPG5 patients in our cohort exhibited significant cervical and thoracic spinal cord atrophy compared with HC. Importantly, thoracic RL total was positively associated with BVMT-R DR and MoCA, explaining 81 and 74% of their variance, respectively. These associations remained after adjusting for BPF, indicating that, in this sample, the relationships between spinal morphology and cognition are not fully explained by macrostructural brain atrophy. In parallel, AD work has reported cervical (C2-C3) cord atrophy relative to HC, with cord atrophy explaining 13% of MMSE variance (27).

Studies of non-progressive spinal cord disorders indicate that, in spinal cord injury (SCI), resting-state functional connectivity between sensorimotor and cognitive/visual/auditory cortices increases and evolves over time after injury (28), suggesting central reorganization/compensation along long-tract-cortical pathways that may influence cognitive phenotypes. SPG5 is predominantly a long-tract demyelinating condition. Multimodal imaging has shown decreased FA and increased RD in both brain and cord on DTI, elevated parieto-occipital mIns/Cr on MRS, and prolonged spinal T1, findings consistent with demyelination-dominant white-matter microstructural injury (29, 30). In addition, involvement of the corticospinal tract/posterior columns and optic radiations has been reported in SPG5 (29). Accordingly, spinal morphometric changes may not only reflect local pathology but also serve as a surrogate marker of broader central network involvement. Moreover, multiple SCI studies indicate that cognitive impairment is not uncommon (31). In one cohort of 83 patients, nearly half scored below the MoCA cutoff (32).

In SPG5, cervical AP total correlated negatively with disease duration, suggesting progressive narrowing of the cervical anteroposterior diameter with longer duration. After adjusting for duration, thoracic RL total remained positively associated with BVMT-R delayed recall but not with MoCA, whereas thoracic CSA total remained positively associated with MoCA. These findings suggest a possible correspondence between cognitive domains and specific morphometric indices. Similarly, studies in adrenomyeloneuropathy (AMN) have reported progressive thoracic cord atrophy and multidomain cognitive impairment (visual memory, spatial cognition, memory, executive functions) (33–36). They also show that MTw abnormalities of the dorsal columns are associated with clinical disability and sensory/postural dysfunction (37), although direct links between quantitative spinal metrics and cognition have not yet been established in AMN.

Previous studies have reported on the neurological basis of cognitive impairment in HSPs. A study analyzed cognitive function and MRI brain structure in 29 HSPs patients, finding a positive correlation between BPF and multiple cognitive assessment scores, including Digit span and Block span (working memory), California Verbal Learning Test, German version, (MVGT, declarative memory), Coloured Progressive Matrices (CPM, executive functions), SDMT (attention), and MMSE (38). In our study, we focused on the relationship between five cognitive domains (language memory, visual memory, visuospatial perception, executive function, and information processing speed) and BPF and CSF volume. Consistent with previous research, we found a significant positive correlation between BPF and MMSE, while no correlation with COWAT. However, we did not observe a correlation between SDMT and BPF in our study. Interestingly, we further analyzed the relationship between CSF and each cognitive domain, revealing a negative correlation between PASAT 3 s and CSF volume.

This work has several limitations. Firstly, the sample size was small, particularly in the MRI portion. Therefore, larger studies are needed to confirm our findings. Secondly, this study only conducted routine MRI examinations on patients, while advanced MRI techniques such as perfusion MRI, diffusion tensor imaging (DTI), functional MRI, among others, can reveal metabolic, microstructural, or functional changes in the brains of HSPs patients. These advanced MRI techniques may provide a more comprehensive assessment of the relationship between a specific cognitive domain and brain damage.

## 5 Conclusion

In summary, patients with SPG5 exhibit cognitive impairment, primarily involving visuospatial perception, executive function, and information processing speed. Thoracic spinal cord morphology (e.g., RL total and CSA total) was associated with aspects of cognitive performance. Future studies should validate these associations in larger, longitudinal cohorts using multimodal imaging and standardized cognitive batteries, and evaluate their potential as clinical research endpoints.

## Data Availability

The raw data supporting the conclusions of this article will be made available by the authors, without undue reservation.
