# Supplementary material for: Association of spinal cord structure with cognition in hereditary spastic paraplegia type 5
Source: Front Neurol. 2026 Jan 2;16:1639011. doi: 10.3389/fneur.2025.1639011 (PMC12807918; doi:10.3389/fneur.2025.1639011)
Supplement: Supplementary file 1 [file Table_1.DOCX]

| **Supplementary Table 1** Psychological test scores of HSPs and HC | | | | | |
| --- | --- | --- | --- | --- | --- |
| **Cognitive tests** | **HSPs, median (IQR)** | | | **HC**  **(N = 47)** | ***P*-value^a^** |
|  | **Total**  **(N = 24)** | **SPG5**  **(N = 10)** | **other-HSPs**  **(N = 14)** |  |  |
| HAMD | 4.5 (2.3-8.5)^b^ | 4.0 (1.0-9.0)^d^ | 6.0 (2.5-8.0)^g^ | 3.5 (1.3-6.8)^i^ | 0.618 |
| HAMA | 5.5 (2.5-7.8)^b^ | 4.0 (2.0-6.0)^d^ | 6.0 (3.5-8.0)^g^ | 3.0 (0.0-5.0)^i^ | 0.390 |
| Fatigue scale | 5.0 (3.0-8.0)^b^ | 3.0 (2.8-5.8)^e^ | 5.5 (3.5-8.8)^*, h^ | 5.0 (2.0-6.0)^j^ | 0.140 |
| PSQI | 5.0 (3.0-6.0)^b^ | 4.0 (3.0-6.0)^d^ | 5.0 (3.0-6.0)^g^ | 3.0 (2.0-5.0)^i^ | 0.877 |
| ESS | 3.0 (2.0-7.0)^c^ | 3.0 (1.8-3.8)^*, f^ | 4.0 (1.5-7.5)^g^ | 5.0 (4.0-8.0)^k^ | 0.467 |
| Abbreviation: HSPs, Hereditary Spastic Paraparesis; SPG5, spastic paraplegia type 5; HC, Healthy Control; HAMD, Hamilton Depression Scale; HAMA, Hamilton Anxiety Scale; PSQI, Pittsburgh Sleep Quality Index; ESS, Epworth Sleeping Scale; IQR, interquartile range.  Other-HSPs refer to genetically confirmed other HSPs subtypes except SPG5, include SPG4, SPG11, SPG30 and SPG76.  Analyses were undertaken to examine differences in HAMD, HAMA, Fatigue scale, PSQI and ESS among the HSPs total, SPG5, and other-HSP groups relative to the HC, signified by * for *P* < 0.05.  ^a^SPG5 vs others; ^b^n = 20; ^c^n = 19; ^d^n = 17; ^e^n = 14; ^f^n = 18; ^g^n = 11; ^h^n = 12; ^i^n = 23; ^j^n = 26; ^k^n = 24  Statistical difference was determined by Mann-Whitney U and independent samples t-test. | | | | | |

| **Supplementary Table 2** Correlation between BVMT-R DR or MoCA and cerebral volume in SPG5 | | | | |
| --- | --- | --- | --- | --- |
| **Cerebral volume** | **BVMT-R DR** | | **MoCA** | |
|  | **Correlation coefficient (*r*)** | ***P* value** | **Correlation coefficient (*r*)** | ***P* value** |
| TIV | 0.696 | 0.124 | 0.546 | 0.262 |
| GMV | 0.612 | 0.196 | 0.223 | 0.671 |
| WMV | 0.675 | 0.141 | 0.551 | 0.257 |
| CSF | 0.365 | 0.477 | 0.712 | 0.112 |
| BPF | 0.354 | 0.491 | -0.317 | 0.541 |
| Abbreviation: TIV, total intracranial volume; GMV, grey matter volume; WMV, white matter volume; CSF, Cerebrospinal fluid; BPF, brain parenchymal fraction.  *P* values are from two-sided Pearson correlation tests. | | | | |

| **Supplementary Table 3** Correlation between BVMT-R DR or MoCA and Spinal cord morphometry in SPG5 | | | | |
| --- | --- | --- | --- | --- |
| **Spinal cord morphometry** | **BVMT-R DR** | | **MoCA** | |
|  | **Regression coefficient (95% CI)** | ***P* value** | **Regression coefficient (95% CI)** | ***P* value** |
| Thoracic spinal cord^a^ |  |  |  |  |
| CSA total | 0.059 (0.021 to 0.096) | 0.015 | - | - |
| RL total | 0.218 (0.068 to 0.367) | 0.019 | 0.329 (0.105 to 0.554) | 0.019 |
| Thoracic spinal cord^b^ |  |  |  |  |
| CSA total | 0.060 (0.020 to 0.100) | 0.017 | - | - |
| RL total | 0.220 (0.109 to 0.332) | 0.008 | 0.320 (-0.009 to 0.649) | 0.053 |
| Abbreviation: CSA, cross-section area; RL, right to left diameter of spinal cord; BVMT-R DR, Brief Visuospatial Memory Test -revisited delayed recall; MoCA, Montreal Cognitive Assessment; CI, confidence interval; BPF, brain parenchymal fraction.  ^a^Adjusted by BPF.  ^b^Adjusted by disease duration. | | | | |

| **Supplementary Table 4** Correlation between disease duration and spinal cord cross-sectional morphometry in patients with SPG5 | | |
| --- | --- | --- |
| **Spinal cord morphometry** | **Disease duration** | |
|  | **Correlation coefficient (*r*)** | ***P* value** |
| Cervical spinal cord |  |  |
| CSA total | -0.543 | 0.266 |
| AP total | -0.886 | **0.019** |
| RL total | -0.086 | 0.872 |
| Thoracic spinal cord |  |  |
| CSA total | 0.029 | 0.957 |
| AP total | 0.200 | 0.704 |
| RL total | -0.086 | 0.872 |
| Abbreviation: CSA, cross-section area; AP, anterior to posterior diameter of spinal cord; RL, right to left diameter of spinal cord.  *P* values are from two-sided Pearson correlation tests. | | |

# Supplementary Figure 1


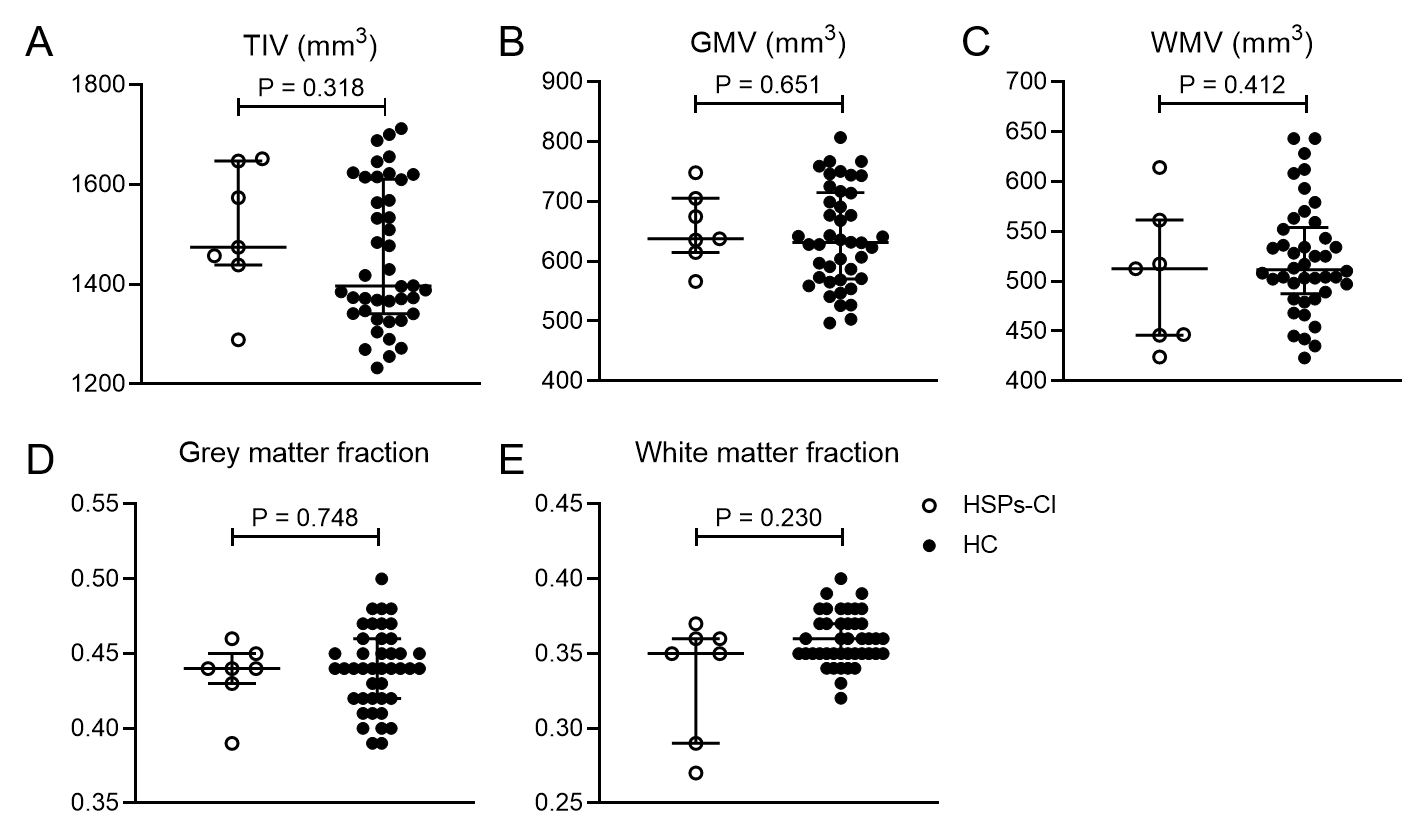


**Supplement Figure 1** **Changes in cerebral volume in HSPs-CI patients. A-E** TIV (**A**), GMV (**B**), WMV (**C**), grey matter fraction (**D**) and white matter fraction (**E**) in HC (n=42, solid circles) and HSPs-CI patients (n=7, hollow circles). Mann–Whitney U and independent samples t-test were used to compare continuous variables between HSPs-CI and HC. The bars represent the median with IQR. HSPs-CI, hereditary spastic paraparesis-cognitive impairment; HC, Healthy Control; TIV, total intracranial volume; GMV, grey matter volume; WMV, white matter volume.
